# Reviewers in 2017

**DOI:** 10.1098/rsob.180027

**Published:** 2018-03-07

**Authors:** David Moore Glover

**Affiliations:** Editor-in-Chief

The Editors and editorial office of *Open Biology* would like to thank all reviewers who volunteered their time and expertise to referee manuscripts published in 2017.

To provide an opportunity for recognition, we list the names of all reviewers who opted in. This article is made permanent and citeable by its digital object identifier (DOI). We encourage you to quote your contribution in your applications for tenure and grants or other forms of research assessment. Where you have provided it, we have included your ORCID so your contribution can be unambiguously assigned to you.

With this in mind, we encourage the use of services such as *Publons*, which is integrated with *Open Biology*, and provides official recognition for your contribution to peer review.

Lujambio A
Abarca-Rojano E
Allen M
Alsina B
Artemyev N
Baines R
Baldini N
Barford D
  http://orcid.org/0000-0001-8810-950X
Barrett J
Basto R
Bauer D
Beggs J
Bertelli C
Bétermier M
Bird A
Black B
Booth W
Bovolenta P
Brochet M
Broer S
Toth A
Bruser T
Caceres A
Caffrey P
Cai Z
  http://orcid.org/0000-0002-9552-4020
Cairns M
Calin G
Wehrle-Halle B
Cavodeassi F
Ceravolo G
Charest P
Cheng T-l
Chick J
Clarke J
Coombe D
Coppola V
  http://orcid.org/0000-0001-6163-1779
Coutard B
Muanprasat C
Dandekar A
Davis T
  http://orcid.org/0000-0003-4797-3152
Deem M
  http://orcid.org/0000-0002-4298-3450
Delitala M
Denapaite D
Dermadi D
Dias MB
Dolezal P
Du M
Duronio B
Edsinger E
Elphick M
Eyers P
Fairbanks D
Fisk H
Fradet-Turcotte A
Frasca A
Fukada S-i
Funabiki H
  http://orcid.org/0000-0003-4831-4087
Gall J
Gamper A
Gao X
Gaponenko V
Garattini E
Mazza D
Gergely F
Giansanti MG
  http://orcid.org/0000-0002-6753-7262
GIBERT Y
Ginger M
  http://orcid.org/0000-0002-9643-8482
Gonzalez C
  http://orcid.org/0000-0002-5259-3608
Gorrell R
Green J
  http://orcid.org/0000-0002-6102-2620
Grundmann M
Guilhot C
GUO G
Hallermann S
Han C
Hantschel O
Harwood A
Hasan G
Helariutta Y
Henry C
Hiraoka Y
Manning G
Schütz G
Homberg U
Hornemann T
Huang X
Iomini C
Jékely G
von Gersdorff H
Joshi A
Joshi P
Kampa B
Kang Z
Katz P
Kawakami Y
Keene A
Kiecker C
Kim L
Nachman I
klosgen RB
Kong Q-P
  http://orcid.org/0000-0002-6046-4494
König B
Kops G
Kurumizaka H
  http://orcid.org/0000-0001-7412-3722
Lafanechère L
Landsberger N
  http://orcid.org/0000-0003-0820-3155
Lavazec C
Lehner C
Lens S
Lim XH
Liu J
Liu S-J
Liu Y
Lopez-Contreras A
Lopez-Domenech G
Lorenzi T
Louis Ed
Lu X
Macdonald A
MacLean L
Gibert J-M
  https://orcid.org/0000-0002-1579-0266
Marcucci L
Martelli C
Martin F
McKee B
McMillan D
  http://orcid.org/0000-0001-6614-4494
González J
  https://orcid.org/0000-0001-7374-9256
Methner A
  http://orcid.org/0000-0002-8774-0057
Winklhofer K
  https://orcid.org/0000-0002-7256-8231
Millar K
Mitsiadis T
Mizuno K
Mollen K
Molnar A
Morris S
Harrington L
  http://orcid.org/0000-0002-4977-2744
Nagasaka K
Nagy L
Nevels M
Nickerson J
Nilsson J
Niwa R
Obha S
Oria M
Osna NA
Overton P
Paulsen O
Pedersen L
Shahbazi M
Pelletier L
  http://orcid.org/0000-0003-1171-4618
Penninger J
Shao M
Piekorz R
Pieters J
Pines J
Pirez N
Poluektova L
Poole A
QIAN S
Raz E
Rehmann H
Renshaw S
Rintoul R
Rippo MR
Roig I
Royle S
  http://orcid.org/0000-0001-8927-6967
Sa J
Salakova M
Sanchez-Buso L
Sao Leao R
Sauer J-D
Scharff C
Schornack S
Selzer M
Silbiger VN
Simian M
Slot J
Smagghe G
Smania AM
Song C
Soultanas P
Sousa M
Sprecher S
  http://orcid.org/0000-0001-9060-3750
Stanton R
Stengl M
Storchova Z
Strittmatter S
Stumph W
Sugita M
Swanson M
Taylor P
Tewari R
Thelen M
Tomida J
Tracey WD
Tyler K
  http://orcid.org/0000-0002-0647-8158
van Echten-Deckert G
Vassar B
Vernes S
Viboud C
Volkman B
von Einem J
Megraw T
Wang D
Wang Q
Wang Y
Wegener C
Géli V
Wieler L
Witcher M
Wright C
Wyllie D
Yang J
Yashiroda H
Yi C
Yu Y
Zachos N
Žárský V
Zernicka-Goetz M
Zhang Q
Zikova A
Zou Z
Zuccolo A

